# A Two-Dimensional Micro Scanner Integrated with a Piezoelectric Actuator and Piezoresistors

**DOI:** 10.3390/s90100631

**Published:** 2009-01-23

**Authors:** Chi Zhang, Gaofei Zhang, Zheng You

**Affiliations:** State Key Laboratory of Precision Measurement Technology and Instruments, Department of Precision Instruments and Mechanology, Tsinghua University, Beijing 100084, P.R. China

**Keywords:** Micro-optical-electro mechanical system, Two-dimensional scanning, Micro scanner, Piezoelectric actuator, Piezoresistor

## Abstract

A compact two-dimensional micro scanner with small volume, large deflection angles and high frequency is presented and the two-dimensional laser scanning is achieved by specular reflection. To achieve large deflection angles, the micro scanner excited by a piezoelectric actuator operates in the resonance mode. The scanning frequencies and the maximum scanning angles of the two degrees of freedom are analyzed by modeling and simulation of the structure. For the deflection angle measurement, piezoresistors are integrated in the micro scanner. The appropriate directions and crystal orientations of the piezoresistors are designed to obtain the large piezoresistive coefficients for the high sensitivities. Wheatstone bridges are used to measure the deflection angles of each direction independently and precisely. The scanner is fabricated and packaged with the piezoelectric actuator and the piezoresistors detection circuits in a size of 28 mm×20 mm×18 mm. The experiment shows that the two scanning frequencies are 216.8 Hz and 464.8 Hz, respectively. By an actuation displacement of 10 μm, the scanning range of the two-dimensional micro scanner is above 26° × 23°. The deflection angle measurement sensitivities for two directions are 59 mV/deg and 30 mV/deg, respectively.

## 1. Introduction

With the development of the micro-electronics technology, micro-optical-electro mechanical systems (MOEMS) have provided new features to spacecraft and the micromation of satellites has become a general trend. For optical scanning and space detection, the laser scanning technique is an active way to detect objects and measure both range and orientation [1, 2]. The two-dimensional micro scanner has great advantages over the conventional scanning mechanisms of the traditional satellites such as low power consumption, small volume and high frequency. It has a broad range of space applications in micro-satellites. At present, Si-based MOEMS is the main technology for micro scanners and many researchers are concentrating on the multidimensional deflection and actuation methods. By the electrostatic, magnetic, or piezoelectric actuation, etc, the micro scanner is capable of scanning a light beam multi-dimensionally with a large scanning angle and low voltage [3–6]. However, many of them have disadvantages such as complicated structures and difficult fabrication processes, both of which result in low reliability and limited applications. Furthermore, micro scanners for space detection and object location require the measurement of deflection angles with high sensitivities. The existing methods are adopting a magnetic mirror with a micro shutter array [1], a piezoresistor with signal filters [2] and two micro mirrors with a PSD sensor [7] for position detection. However, the mechanisms and circuits of these are complicated, with low integration and limited sensitivity.

Aiming at fixing these deficiencies, a compact two-dimensional micro scanner with a piezoelectric actuator and piezoresistors is presented in this paper. It has a small volume with high integration and a decoupling measurement method of deflection angles with high sensitivities is presented for the two-dimensional micro scanner. The details concerning the structure, operation principle, simulation, deflection angles measurement, fabrication process and experimental results are described.

## 2. Two-Dimensional Micro Scanner

### 2.1. Structure and Principle

**Figure 1.**
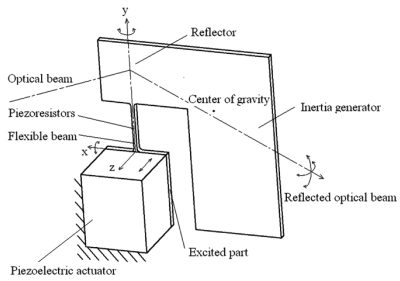
Structure of two-dimensional micro scanner.

A two-dimensional micro scanner with a piezoelectric actuator and piezoresistors is designed as shown in Figure 1. The micro scanner structure consists of a reflector, an inertia generator, a flexible beam and an excited part. The reflector and inertia generator are formed together, and linked with the excited part by the flexible beam. The excited part is connected to the piezoelectric actuator. The piezoresistors are integrated on the surface of the flexible beam for deflection angle decoupling measurement.

**Figure 2.**
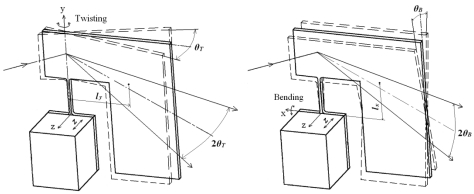
Two resonance vibration modes.

The piezoelectric actuator deforms along z-axis by pulsant driving voltage and the excited part vibrates in the z-axis. Because the center of gravity of the reflector and inertia generator is away from each rotational axis (x and y), the scanner has two resonance vibration modes: twisting around the y-axis and bending along the x-axis, as shown in Figure 2. The two-dimensional micro scanner is thus equivalent to a two dimensional vibration system with two different resonant frequencies. Actuating the scanner at each resonant frequency can make the scanner vibrate with large deflection angles *θ_T_* and *θ_B_* around the y-axis and x-axis, respectively. When a resultant voltage including two different resonant frequencies is imposed to the piezoelectric actuator, both vibration modes are excited and the micro scanner is capable of scanning a light beam two-dimensionally with large scanning angles with a single driving source.

### 2.2 Modeling and simulation

In the two-dimensional micro scanner structure, the reflector and inertia generator can be considered as a single mass at the end of the flexible beam. Accordingly, the deformations in two directions are equivalent to the twisting and bending of the flexible beam. The two-dimensional micro scanner is driven by the actuation of the piezoelectric actuator in z-axis and the flexible beam is twisted and bent by the inertial force. Therefore, the movements of the micro scanner can be equivalent to the forced vibration of the system based movement. Under micro deformation circumstances, the movements of the micro scanner can be described as a “mass-spring-dashpot” system. For the twisting and bending of the flexible beam in the two different modes of the micro scanner, each movement is independent from the other. Therefore, the movement equations of the 2-DOF model can be represented by equation (1):

(1) $$I_{n}\;{\overset{¨}{\theta }}_{n}+C_{n}\;{\dot{\theta }}_{n}+K_{n}\;\theta_{n}=M_{n},$$

where *I_n_* is the inertial momentum, *C_n_* is the damping coefficient and *K_n_* is the spring stiffness. *M_n_* is the generated moment of the piezoelectric actuator and *θ_n_* is the deflection angle of the reflector. The resonance frequencies *ω_n_* for each vibration mode are described by equation (2):

(2) $$\omega_{n}=\sqrt{\frac{K_{n}}{I_{n}}}.$$

When one resonance frequency is far from the other, the steady-state response of the 2-DOF system can be approximated as follows:

(3) $$\theta_{n}(t)=B_{n}\;\sin(\omega_{n}t-\varphi_{n}),$$

where *B_n_* is decided by the structure of the scanner and the amplitude of the piezoelectric actuator. When *n*=1, the equation is concerned with the twisting mode and when *n*=2, the equation is concerned with the bending mode. Under the circumstances of micro damping, the two resonant amplitudes of the system, that are the maximal deflection angles in two directions are described as follows [8]:

(4) $$\theta_{1-\max}=\frac{z}{2l_{y}\xi_{1}},$$

(5) $$\theta_{2-\max}=\frac{3z}{4l_{x}\xi_{2}},$$

where *z* is the actuation displacement of the piezoelectric actuator, *l_y_* is the center of gravity to the y-axis distance, *l_x_* is the center of gravity to the x-axis distance, *ξ_1_* and *ξ_2_* are the damping ratios of the 2-DOF vibration system.

Accordingly, if the difference between the resonant frequencies of the scanner is large, the effect of the imposed external actuation which results in the resonance in one direction can be ignored in the other direction. When the actuations include both resonant frequencies, the micro scanner can achieve resonance simultaneously in both directions with large amplitudes. From the theoretical inference, the maximal deflection angles in two directions are proportional to the actuation displacement of the piezoelectric actuator.

**Figure 3.**
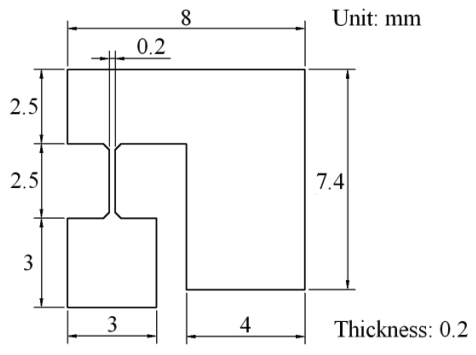
Dimensions of two-dimensional micro scanner.

**Figure 4.**
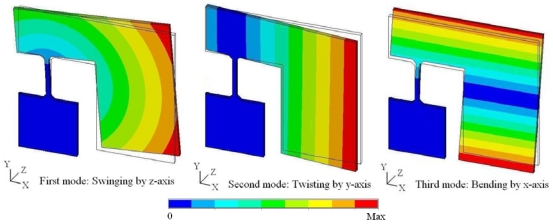
Three modes by FEM modal simulation.

Figure 3 shows the dimensions of the micro scanner. FEM simulation is used for the verification of the structure and modeling with ANSYS software. The silicon material is adopted and the analysis results of low order modal simulation are shown in Figure 4. The first mode is swinging by z-axis at 202.5Hz, the second mode is twisting by y-axis at 229.6Hz and the third mode is bending by x-axis at 473.2Hz when the damping is ignored. The actual resonance frequencies *ω_nm_* are influenced by the damping, which are described as follows:

(6) $$\omega_{nm}=\sqrt{1-2{\xi_{n}}^{2}}\cdot \omega_{n}.$$

**Figure 5.**
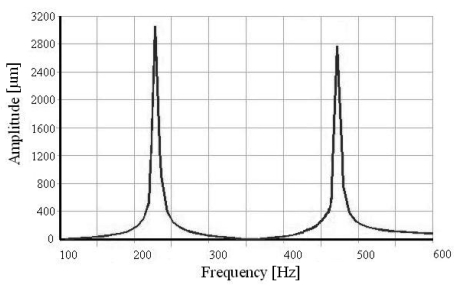
FEM Harmonic Response Simulation.

The micro scanner is then analyzed by the harmonic response simulation. Since the piezoelectric actuator deforms along the z-axis at a certain frequency, the reflector is driven by a harmonic inertial force along the z-axis. The movement of the reflector with the changed driving frequency is shown in Figure 5.

The simulation results indicate that the first mode will not appear when the micro scanner is actuated along the z-axis, while twisting around the y-axis and bending by the x-axis with large deflection angles could be achieved at the respective resonant frequencies. The difference between the two resonant frequencies is large, which meets the design requirement. Furthermore, the maximal deflection angles are linear to the inertial force which is proportional to the actuation displacement of the piezoelectric actuator [8]. Therefore, the simulation analysis verifies that the design is effective and the modeling is correct.

## 3. Deflection angles measurement

### 3.1 Principle and method

Deflection angle sensing is based on the piezoresistive effect which has the advantages of favorable dynamic characteristics and high sensitivities. The stresses in the longitudinal, transverse and tangential directions of the piezoresistor cause the change of the resistance. The piezoresistive effect in plane is described as follows [9]:

(7) $$\frac{\Delta R}{R}=\pi_{l}\sigma_{l}+\pi_{t}\sigma_{t}+\pi_{\tau }\sigma_{\tau },$$

where *σ_l_* is the longitudinal stress, *σ_t_* is the transverse stress and *σ_τ_* is the tangential stress. *π_l_* is the longitudinal coefficient, *π_t_* is the transverse coefficient and *π_τ_* is the tangential coefficient.

**Figure 6.**
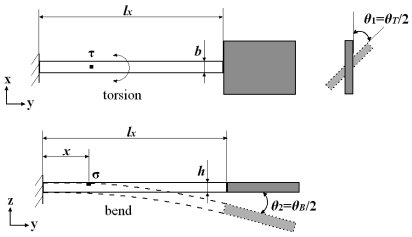
Surface stresses on flexible beam.

Piezoresistors are laid on the flexible beam for the measurement of the deflection angles for two vibration modes. The micro scanner twisting around y-axis and bending of the x-axis are equivalent to the torsion of a rod and the bend of a cantilever beam with a mass at the end, respectively. The surface stresses generated on the flexible beam are shown in Figure 6. The shear stress *τ* and the normal stress *σ* at distance *x* away from the end of the flexible beam can be described as follows:

(8) $$\tau =\frac{\beta bG}{\alpha l_{x}}\theta_{T},$$

(9) $$\sigma =\frac{\text{xhE}}{2{l_{x}}^{2}}\theta_{B},$$

where *b* is the width of the flexible beam, *h* is the thickness of the flexible beam, *E* is the Young's modulus of silicon and *G* is the shear modulus of silicon.

**Figure 7.**
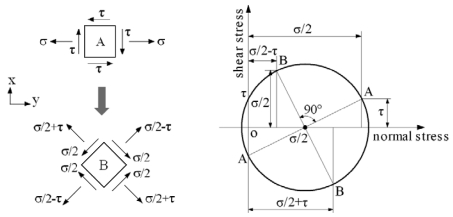
Stress states of coupled motions.

When the scanner is scanning two-dimensionally, the motions of the flexible beam are coupled with both the torsion and bend. The surface stress states of a cell A at distance *x* away from the end of the flexible beam are analyzed as shown in Figure 7. The cell A simultaneously bears the shear stress *τ* and the normal stress *σ* [10]. When a piezoresistor is oriented along the axis direction, the longitudinal stress is *σ*, the transverse stress is zero and the shear stress is *τ*. According to the Mohr's circle for plane stress transformation, the stress states of a cell B on the flexible beam are shown in Figure 7. When a piezoresistor is oriented along 45 degrees from the axis direction, the longitudinal stress is *σ*/2+*τ*, the transverse stress is *σ*/2-*τ* and the shear stress is *σ*/2.

Therefore, the change of the resistance in the piezoresistor is influenced by both motions and not proportional to either deflection angle. The piezoresistor design of appropriate directions and crystal orientations can realize the decoupling measurement for two deflection angles, which will be presented below with detailed information.

### 3.2 Piezoresistor design

The change of the resistance in piezoresistor is related to not only the longitudinal, transverse and tangential stresses, but also the piezoresistive coefficients in longitudinal, transverse and tangential directions. These piezoresistive coefficients depend strongly on the crystal orientation and doping type, which are described as follows [9]:

(10) $$\pi_{l}=\pi_{11}-2\left(\pi_{11}-\pi_{12}-\pi_{44}\right)\;\left(l_{1}^{2}m_{1}^{2}+l_{1}^{2}n_{1}^{2}+m_{1}^{2}n_{1}^{2}\right),$$

(11) $$\pi_{t}=\pi_{12}+\left(\pi_{11}-\pi_{12}-\pi_{44}\right)\;\left(l_{1}^{2}l_{2}^{2}+m_{1}^{2}m_{2}^{2}+n_{1}^{2}n_{2}^{2}\right),$$

(12) $$\pi_{\tau }=-2\left(\pi_{44}+\pi_{12}-\pi_{11}\right)\;\left(l_{1}^{3}l_{2}+m_{1}^{3}m_{2}+n_{1}^{3}n_{2}\right),$$

where *π_ij_* is the piezoresistance tensor. (*l*_1_, *m*_1_, *n*_1_) and (*l*_2_, *m*_2_, *n*_2_) are the sets of direction cosines of the longitudinal piezoresistor orientation and the transverse piezoresistor orientation in the crystal axis.

**Figure 8.**
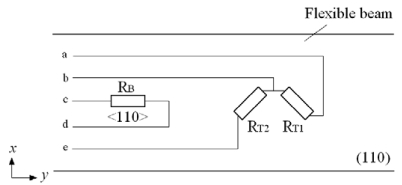
Directions and crystal orientations of piezoresistors.

**Figure 9.**
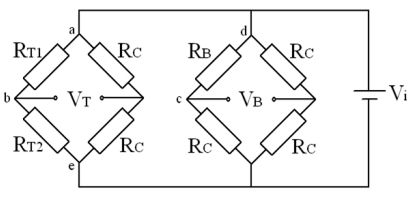
Piezoresistors connection of two Wheatstone bridges.

According to the general expressions for the piezoresistive coefficients, an n-type silicon substrate in (110) wafer is selected. In order to extract the shear stress *τ* and the normal stress σ respectively and counteract the influence by each other, two p-type silicon piezoresistors *R_T_*_1_ and *R_T_*_2_ are oriented along ±45 degrees off y-axis and a p-type silicon piezoresistor *R_B_* is oriented along y-axis in <110> crystal orientation. The directions and crystal orientations of piezoresistors and the connection of two Wheatstone bridges are shown in Figure 8 and Figure 9. Large piezoresistive coefficients are obtained for the high measurement sensitivities, which are shown in Table 1.

With the equation (7) and the stress states analysis in Figure 6, the piezoresistive effects are described as follows:

(13) $$\frac{\Delta R_{T1}}{R_{T1}}=\pi_{lT}(\frac{\sigma_{T}}{2}+\tau_{T})+\pi_{tT}(\frac{\sigma_{T}}{2}-\tau_{T})+\pi_{\tau T}\frac{\sigma_{T}}{2},$$

(14) $$\frac{\Delta R_{T2}}{R_{T2}}=\pi_{lT}(\frac{\sigma_{T}}{2}-\tau_{T})+\pi_{tT}(\frac{\sigma_{T}}{2}+\tau_{T})+\pi_{\tau T}\frac{\sigma_{T}}{2},$$

(15) $$\frac{\Delta R_{B}}{R_{B}}=\pi_{lB}\sigma_{B}+\pi_{\tau B}\tau_{B},$$

where *σ_T_* and *τ_T_* are the normal stress and the shear stress on piezoresistors *R_T_*_1_ and *R_T_*_2_ while *σ_B_* and *τ_B_* are the normal stress and the shear stress on piezoresistor *R_B_*. With the obtained piezoresistive coefficients, the output voltages *V_T_* and *V_B_* of the two Wheatstone bridges are respectively obtained as follows:

(16) $$V_{T}=\frac{V_{i}}{4}\cdot (\frac{\Delta R_{T1}}{R_{T1}}-\frac{\Delta R_{T2}}{R_{T2}})=69.1\times (10^{-2}/\text{GPa})\cdot \tau_{T}\cdot V_{i},$$

(17) $$V_{B}=\frac{V_{i}}{4}\cdot \frac{\Delta R_{B}}{R_{B}}=18.0\times (10^{-2}/\text{GPa})\cdot \sigma_{B}\cdot V_{i},$$

where *V_i_* is the input voltage of the two Wheatstone bridges. The *τ_T_* is proportional to the deflection angle *θ_T_* and the *σ_B_* is proportional to the deflection angle *θ_B_* according to the equation (8) and (9). Therefore, there are linear relationships between output voltages of the two Wheatstone bridges and deflection angles in two directions, respectively. The twisting and bending of the flexible beam is completely decoupled and the deflection angles of each direction are obtained independently and precisely.

## 4. Fabrication and package

### 4.1 Fabrication process

**Figure 10.**
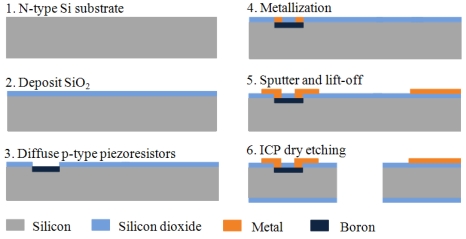
Fabrication process flows.

**Figure 11.**
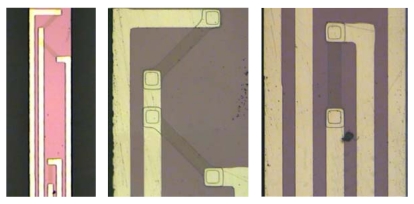
Micrographs of flexible beam and piezoresistors.

The fabrication process flows are shown in Figure 10. The two-dimensional micro scanner is fabricated using a bulk silicon process, starting with an n-type silicon substrate of 300 μm thickness. Boron doping produces p-type piezoresistors on the surface. In order to obtain the desired resistivity r_0_ = 1.1×l0^-2^ Ω·cm, the dimensions of the piezoresistors are set to 100 μm × 10 μm with 0.5 μm depth and the boron ion implantation density is 8.0×10^18^ ions/cm^2^ at the temperature of 1,100°C with 11 minutes duration. The sputter and lift-off process was adopted and golden thin film lines with width of 10 μm are connected and laid on the flexible beam. The micrographs of the flexible beam and piezoresistors are shown in Figure 11. Finally, the micro scanner structure is released by inductive coupled plasma (ICP) dry etching. In addition, the piezoelectric actuator is fabricated by the precision machining and connected to the excited part by the epoxy resins.

### 4.2 Package of the micro scanner

The two-dimensional micro scanner is excited by the piezoelectric actuator which is connected to the excited part with epoxy resin. There are two circuit boards linked from top to bottom. The piezoelectric actuator is connected to the bottom board and the piezoresistors are connected to the top board with golden wire bonding. The detection signal of the deflection angles are output from the bottom board on which the detection circuits are operated.

**Figure 12.**
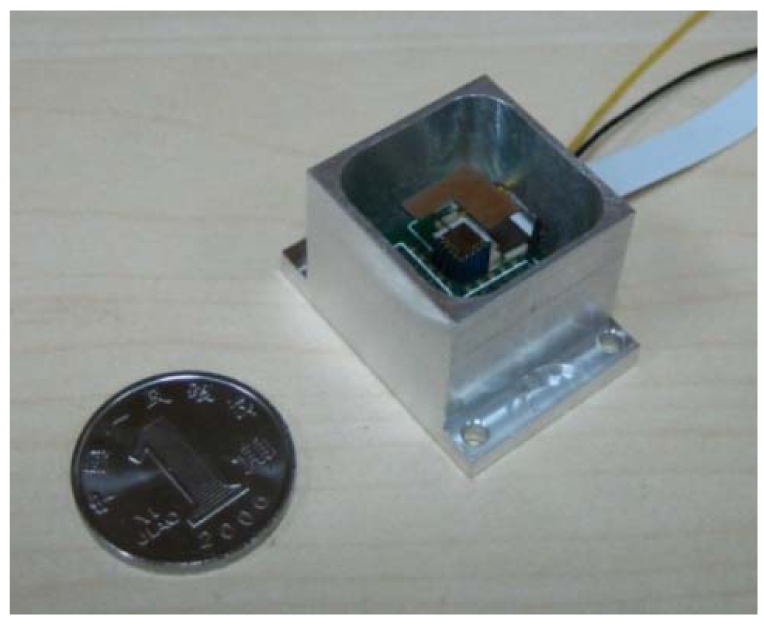
Package of two-dimensional micro scanner.

The micro scanner is packaged and protected by a stainless steel case. The top of the package is open for the reflector and closed with a translucent optical glass. The actuation and detection signal are imported and exported through a window in the side. The micro scanner, the piezoelectric actuator and the piezoresistors detection circuits are integrated in the unit module with the size of 28 mm × 20 mm × 18 mm, as shown in Figure 12.

## 5. Experimental Results

### 5.1 Scanning characteristics

**Figure 13.**
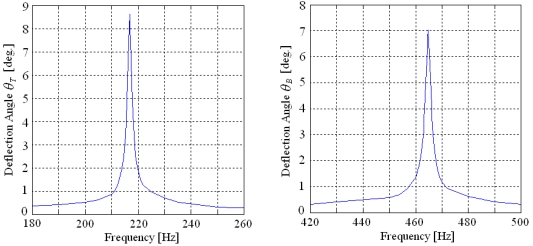
Scanning amplitude frequency responses in two directions.

The two resonance frequencies of the two-dimensional micro scanner are measured by the frequency sweeping method and the scanning amplitude is detected by a laser interferometer measurement system. With an actuation displacement of 3.6 μm, the scanning amplitude-frequency responses in two directions are shown in Figure 13. The resonance frequencies are 216.8 Hz and 464.8 Hz, respectively. Both of them are slightly less than the FEM modal simulation results for the existence of the damping. The deflection angles twisting by y-axis and bending by x-axis are 8.6°×7.0°. The frequency sweeping method verifies the operation principle and the resonance frequencies are closed to the simulation results with a litter difference because of damping.

**Figure 14.**
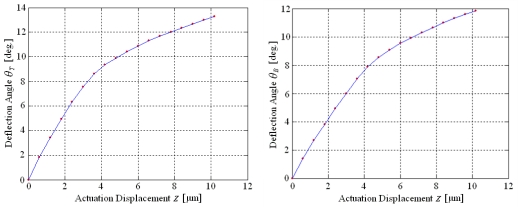
Deflection Angles Characteristics in Two Directions.

The relationships of each deflection angle and the actuation displacement in the resonance modes are shown in Figure 14.

**Figure 15.**
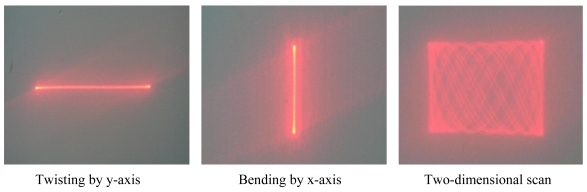
Scan Patterns of Two-Dimensional Micro Scanner.

The experimental results indicate that there are linear relationships between each deflection angle and the actuation displacement when the actuation displacement is less than 4 μm, which is in agreement with the theoretical equations (4) and (5). Nevertheless, the curves are gradually smoothed when the actuation displacement is more than 4 μm. The primary reason is that the deflection angles increase with the increasing actuation displacement which has overstepped the micro deformation condition in the theoretical model. By an actuation displacement of about 10 μm, the deflection angles twisting along the y-axis and bending on the x-axis are 13.3° × 11.8°. By reflecting the optical beam, the scanning range of the two-dimensional micro scanner is above 26° × 23°. The scan patterns of the twisting by y-axis, bending by x-axis and two-dimensional scan are shown in Figure 15.

### 5.2 Piezoresistor characteristics

**Figure 16.**
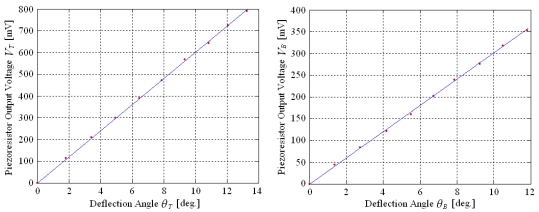
Piezoresistor Output Characteristics in Two Directions.

The deflection angles of the two-dimensional micro scanner are measured by the piezoresistors detection circuits and the piezoresistor characteristics are indicated by the output voltages of the two Wheatstone bridges. The relationships of the corresponding piezoresistor output voltage and each deflection angle in the resonance modes are shown in Figure 16. There are linear relationships between each output voltage and each deflection angle. The deflection angles measurement sensitivities for two directions are 59 mV/deg and 30 mV/deg, respectively.

The piezoresistor output voltage in twisting mode is less than the theoretical calculation while the one in bending mode approaches the theoretical value. This is because that the length of the piezoresistor is close to the width of the flexible beam. The average stress on piezoresistors *R*_*T*1_ and *R*_*T*2_ in practice are less than the maximal shear stress on the surface of the flexible beam in the theoretical calculation. By comparison, the length of the piezoresistor is far less than the flexible beam length. The average stress on piezoresistor *R_B_* in practice approaches to the maximal normal stress on the surface of the flexible beam in the theoretical calculation.

## 6. Conclusions

To achieve two-dimensional laser scanning, a compact two-dimensional micro scanner has been developed in this paper. The micro scanner is excited by a piezoelectric actuator and the piezoresistors are integrated for the deflection angle measurement in two directions. The design, modeling, simulation and fabrication are described and the experimental results are characterized. The unit module including the micro scanner, the piezoelectric actuator and the piezoresistors detection circuits are of a size of 28 mm × 20 mm × 18 mm. The two scanning frequencies are 216.8 and 464.8 Hz for each direction of the micro scanner. With an actuation displacement of about 10 μm, a scanning range above 26° × 23° is obtained. The piezoresistor design has realized decoupling measurements for the bending and twisting deflection angles of the two-dimensional micro scanner. The piezoresistor output sensitivities for the two directions are 59 and 30 mV/deg, respectively. The total power consumption of the piezoelectric actuation and piezoresistors measurement is below 1 W. The two-dimensional micro scanner has the great advantages of low power consumption, small volume, high frequency, large deflection angles and high measurement sensitivities. It is suitable for the micro laser scanning system and has a broad range of potential applications in the field of space target detection and position measurement in micro-satellites.

## Figures and Tables

**Table 1.** The value of the piezoresistive coeffients (10^-2^/GPa).

*π_lT_*	*π_tT_*	*π_τT_*	*π_lB_*	*π_tB_*	*π_τB_*
88.1	-50	-32.6	71.8	-1.1	0
